# A Swine Model of Neural Circuit Electromagnetic Fields: Effects of Immediate Electromagnetic Field Stimulation on Cortical Injury

**DOI:** 10.7759/cureus.43774

**Published:** 2023-08-19

**Authors:** James Brazdzionis, Mohamed M Radwan, Finosh Thankam, Yssel Mendoza Mari, David Baron, David Connett, Devendra K Agrawal, Dan E Miulli

**Affiliations:** 1 Neurosurgery, Riverside University Health System Medical Center, Moreno Valley, USA; 2 Translational Research, College of the Osteopathic Medicine of the Pacific, Western University of Health Sciences, Pomona, USA; 3 Psychiatry and Behavioral Sciences, College of the Osteopathic Medicine of the Pacific, Western University of Health Sciences, Pomona, USA

**Keywords:** electromagnetic field stimulation, neuronal circuit, swine model, controlled cortical impact, stimulation, tbi, emf, sensors, traumatic brain injury, electromagnetic field

## Abstract

Background

Neurologic diseases have profound disability, mortality, and socioeconomic effects worldwide. Treatment of these disorders varies but is largely limited to unique factors associated with neural physiology. Early studies have evaluated alterations in electromagnetic fields (EMF) due to neural disorders with subsequent modulation of EMF as a potential treatment modality. Swine models have begun to be evaluated as translational models in this effect.

Methods

EMF measurements of a Yucatan miniswine were recorded using proprietary non-contact, non-invasive induction sensors with a dual layer Mu-metal and interlaced copper mesh helmet. The swine then underwent controlled cortical impact (CCI) to simulate traumatic brain injury (TBI). Twenty minutes post-injury after surgical wound closure, the swine underwent targeted EMF signal modulation using a signal generator to stimulate the swine's injured cortical circuit using a sinusoidal wave individualized at 2.5 Hz with a 500mV positive offset at 1V. After 10 days of stimulation, settings were modified to another individualized frequency of 5.5 Hz, 500mV positive offset and 1V for stimulation. Behavioral patterns in swine were evaluated, and EMF measurements were recorded daily prior to, during, and after stimulation. Artificial intelligence (AI) models evaluated patterns in EMF signals. Histology of the stimulated swine cortex was evaluated using hematoxylin and eosin staining and pentachrome staining and compared to a control swine without stimulation and a swine that had received stimulation two days post-injury in a delayed fashion. Serial serum specimens and tissue at the time of euthanasia were obtained for assessment of neuron-specific enolase (NSE) concentration.

Results

Pre-operative and post-stimulation measurements demonstrated differences in patterns and activity early on. There was an identified peak at 1.6Hz, not frequently seen pre-operatively. There were convergent frequencies in both data sets at 10.5 Hz and 3.9 Hz. Plateaus and decreased variability of changes in slope were identified early in the post-injury phase. AI modeling identified early similarities in pre-operative and post-stimulation measurements through the patterns of peaks with similarities on postoperative day 10 and similarities in the valleys on postoperative day 17. Histologic specimens identified increased degrees of apoptosis and cellular death in the non-stimulated control compared to the stimulated swine. Similarly, the immediately stimulated swine had less apoptosis and increased histologic viability at the site of injury compared to the two-day delayed stimulation swine. There were increased levels of NSE noted in the stimulated swine at the site of injury compared to non-injured sites and the control swine.

Conclusions

Cortical function was appropriately measured through induction sensors and shielding in the form of a helmet and electromagnetic field channels. Early stimulation resulted in the early and durable recovery of neuronal circuit-driven electromagnetic field patterns. Histology identified increased viability of neurons with fewer apoptotic neurons and glial cells in stimulated swine with early stimulation identifying the best effect compared to a non-stimulated subject. This recovery identifies change and recovery at the circuit, cellular, and subcellular levels that potentiate the need for further study of EMF modulation as a treatment modality in neurological disorders.

## Introduction

Swine models have been researched as translational models for traumatic brain injury (TBI) [1,2]. Within this model, evaluation of neural circuit-driven electromagnetic field (EMF) characteristics has occurred in the non-injured and post-TBI state [3,4]. These models have utilized controlled cortical impact (CCI) to simulate the incident TBI. Interestingly, one study has also investigated the effects of modulation of this EMF through stimulating technologies [4].

From a clinical perspective, the ability to evaluate neuronal circuits and functionality in real-time, non-invasively, would be invaluable in an intensive care unit setting in all neurologic conditions. If clinically correlated, identifying abnormalities in EMF may help guide targeted treatments, prognosis, and appropriate discussions regarding goals of care through the fundamental understanding of incident regions of injury. From a physiologic perspective, neurons participate in signal transduction through chemical and electrical processes. These functions cause changes in neurons nearby, allowing for spatial and temporal summation. Correspondingly, the changes in electrical charge within neurons cause a necessary change in the electromagnetic field due to Faraday's law [5]. Furthermore, as neurons function through electrical and chemical transmission systems to generate action potentials that work in summation to participate in appropriate processes modulating abnormal activity may help promote neurons in non-injured regions with disrupted inputs to participate in their appropriate functions [5,6]. By restoring this EMF environment to an appropriate "normal", there is thought that neuroplasticity may be more likely, and there may even be restorative effects [7]. Correspondingly, in rodent models, it has been identified that transcranial magnetic stimulation (TMS) has been used to treat TBI with encouraging results through histological and physiologic recovery within injured tissues [8,9]. Similarly, TMS has been found to improve post-concussion depression and other symptoms secondary to the injury due to the modulation of cortical and subcortical neural networks [8-13]. Modulating the electromagnetic field has been under early investigation through smaller animal studies in a variety of neurologic disorders, including seizure, stroke, degenerative diseases, and even for differentiation of stem cells [7,14-16]. Within the large animal world, there have been few investigations that have evaluated targeted electromagnetic field stimulation to a region of injury [4]. These studies have differed from TMS due to their targeted nature with stimulation built into the sensing probes [4].

Within the large animal literature, there are few studies that evaluate neural EMF and even fewer that evaluate the effects of targeted and individualized EMF stimulation on modulating cortical pathways. One such study did identify that there were differences within EMF measurements pre-injury, post-injury, and post-injury post-stimulation with a convergence of post-stimulation patterns towards those identified in the pre-operative, normal phase [4]. This study evaluated these effects after a brief waiting period to obtain post-operative measurements. In this post-operative time period, it is possible that irreversible injury and necrosis could have been occurring within the injured neurons and glial cells, reducing the effects of stimulation. In addition, from a clinical perspective, if early stimulation results in the potential for greater healing, this may be an appropriate avenue for future trials to improve outcomes in TBI. Furthermore, that study was additionally a small pilot study with a singular sample to evaluate feasibility. As a result of this, we aimed to evaluate the effects of immediate EMF stimulation post-CCI on a Yucatan mini-pig model and to analyze the reproducibility of the initial trial evaluating effects of stimulation on post-TBI changes in EMF using real-time, non-invasive, non-contact induction sensors and an electromagnetic shielded helmet with EMF channels.

## Materials and methods

The protocol for this study was approved by the Western University of Health Science Institutional Animal Care and Use Committee under protocol number R23IACUC003. The swine model investigated was a Yucatan minipig (Premier BioSource, Ramona, CA). During the investigation, the pig was maintained on a normal diet with unrestricted access to water. Prior to the study, the swine was acclimatized to a helmet analog within its pen in the vivarium. Proprietary induction sensors (BS-1000; Quasar Federal Systems, San Diego, California) with sensitivities of 1pT/rtHz at 1Hz were placed within an EMF channel in a dual-layer Mu-metal (MuMETAL®; Magnetic Shield Corporation, Bensenville, Illinois) with interlaced copper mesh were used to evaluate EMF signals generated by the swine's cortex through previously published protocols [3,4]. The Mu-metal helmet utilized was previously used in similar studies [3,4]. EMF measurements of the subject were recorded daily prior to induction of TBI through a previously published CCI protocol [3]. Data was acquired at a rate of 5-thousand samples per second with a 16-bit National Instruments data card (National Instruments Corporation, Austin, Texas) after filtering with a 2kHz low pass filter with a 10x gain module. Signals were captured between 1 Hz and 2 kHz within cylindrical regions of detection. Igor ® Pro version 8 (WaveMetrics, Lake Oswego, Oregon) was used to analyze data. Fast Fourier transform (FFT) algorithms were employed to acquire data for investigation of the frequency domain. Swine EMF was recorded through the protocol employed by Brazdzionis et al. using published sensor and helmet orientation [3,4]. Accordingly, sensors were located with B319 over the left frontal region, Bx in the left parietal region, By in the right frontal region, and Bz in the right parietal region. When measurements were obtained in all studies, the helmet containing the EMF channels and sensors was held directly over the calvarium without touching the head, ensuring the midline was located over the midline of the snout. Treats were used to train pigs during acclimatization and used during measurements for consistency. Recorded data was evaluated for 20-second consecutive bins where the helmet was maintained in appropriate positioning over the swine's scalp. These 20-second bins containing 100,000 data points per Hz were evaluated. Using this protocol, daily measurements of the subject in the vivarium were taken prior to CCI, preempted by daily assessments of external EMF noise activity within the vivarium. Further, the swine was monitored for baseline behavioral functions defined as prandial activities, ambulation, and phonation consistent with previous investigations [3,4].

Prior to inducing CCI, baseline EMF recordings were obtained of the operating suite, followed by post-induction EMF recordings of the subject. The surgical procedure consisted of a CCI model to induce TBI. A previously published gravity-dependent model was utilized, dependent on an 8.369g steel ball dropped through a three-foot galvanized steel pipe falling at a rate of 3.579 meters/second [3,4]. The incision was planned over the cruciate gyrus using the bregma with a 3-4 cm linear incision. A burr hole was drilled using a Hudson brace for which the steel ball was dropped twice onto the exposed dura and brain to induce TBI. After hemostasis was obtained, bone wax was used to line the site of the burr, and a layered closure was performed. After closure, a postoperative EMF measurement was recorded. Anesthesia remained consistent with previous studies that employed acepromazine, ketamine, telazol, xylazine, propofol, and isoflurane. Standard monitoring occurred of vital signs during the pre-induction, post-induction, pre-impact, and post-impact phases to evaluate changes in blood pressure.

Post-surgery, immediately following the postoperative EMF measurement, an EMF signal generator was connected to sensor B319 to direct a signal towards the left frontal region where the impact was performed. While the subject was still anesthetized, the EMF signal generator was turned on with stimulation occurring for three minutes with a positive 500mV offset with the previously identified signal set at 2.5Hz at 1 V. This initial stimulation trial occurred 20 minutes post injury. This was repeated once for a total of two stimulation trials while under anesthesia. These settings are consistent with settings used in a previous trial [4]. During this stimulation, sensors Bx, By, and Bz were configured to obtain measurements of cortical EMF. After the three minutes elapsed, the signal generator was turned off. Post-stimulation EMF measurements were then taken five minutes post-stimulation using all sensors. The swine was then recovered.

Once ambulatory in its pen, EMF measurements were taken of the subject and recorded. Following this, the swine underwent once-a-day stimulation with the same setting of 2.5 Hz, 500mV positive offset at 1V for 10 sessions with pre-stimulation and post-stimulation EMF measurements. After 11 sessions total, including the two on postoperative day 0 (10 days), the stimulation settings were changed to a previously determined 5.5 Hz 500mV positive offset at 1V the remaining six sessions to evaluate whether the previously utilized stimulation thresholds would produce reproducible changes [4]. During the entire project, the subject was monitored for behavioral changes, which were recorded. No mortality occurred in this subject during the procedure or post-procedure management. After completion of the trial, the subject was euthanized using a solution of pentobarbital and phenytoin per protocol on postoperative day 21. During the study, blood samples were obtained pre-operatively while in the operative suite, post-operatively on the day of surgery, and serially throughout the study during measurements and on the day of sacrifice for analysis of neuron-specific enolase (NSE). After sacrifice, the brain was immediately harvested, tissue samples were obtained, and gross specimen was placed in formalin for sectioning. Samples were evaluated at the site of injury with hematoxylin and eosin (H&E) and pentachrome stains. Blood and pathology samples were additionally obtained from two additional pigs used in two prior studies, with samples obtained along the same timeline with the same sacrifice and harvest techniques. The first additional swine evaluated underwent CCI impact and EMF recordings but was not exposed to EMF stimulation. The second pig underwent the exact protocol as previously published [4]. This second swine was stimulated beginning on postoperative day two [4]. Sample preparation and initial analysis of NSE levels occurred with the assistance of an assistant blinded to the interventions.

EMF measurements were analyzed by two authors (DM and JB). The prominent peaks and valleys in each waveform were identified for sensor B319 in the daily measurements using previously utilized protocol [3,4]. Peaks were defined as the amplitude for an associated frequency where the amplitude of the adjacent frequencies were less than the identified frequency. Valleys were defined as the inverse of this where-in the valley was the minimum amplitude where the adjacent amplitudes were greater than the identified amplitude. Sensor B319 was analyzed due to its directionality over the lesion. Recorded peaks and valleys were input into a large language model (LLM) artificial intelligence (AI) software and queried for pattern identification. Patterns were then reviewed for AI hallucinations.

## Results

Swine EMF analysis

**Figure 1:**
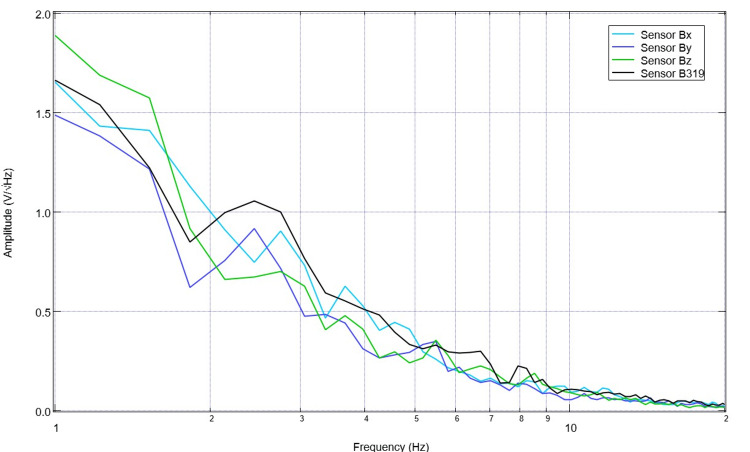
Baseline electromagnetic field measurements of the Yucatan minipig prior to controlled cortical impact Baseline electromagnetic field measurements are noted. Of note, there is a prominent peak at 2.5 Hz and a peak at 5.5 Hz.

Daily EMF recordings were initiated prior to controlled cortical impact to evaluate a pre-injury baseline. As with all measurements, the evaluated EMF signals are from 20-second binned measurements where the helmet was centered over the swine head with minimal movement of the subject. A representative graph of the pre-injury baseline was noted to have peaks at 2.5 Hz, 5.5 Hz, 6.7 Hz, 7.9 Hz, 8.8 Hz, and 10 Hz when evaluating the B319 sensor (Figure 1). Similarly, the valleys measured by the B319 sensor were identified to be 1.8 Hz, 3.2 Hz, 5.2 Hz, 6.1 Hz, 7.4 Hz, 8.5 Hz, and 9.5 Hz. Overall, there were twelve pre-operative measurements evaluated.

**Figure 2:**
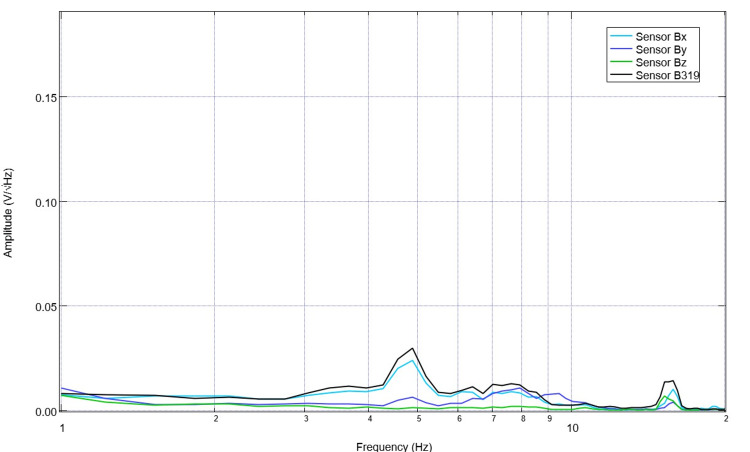
Electromagnetic field measurements of the operating room without a subject within the helmet Electromagnetic field measurements were obtained in the operating room without a subject.

**Figure 3:**
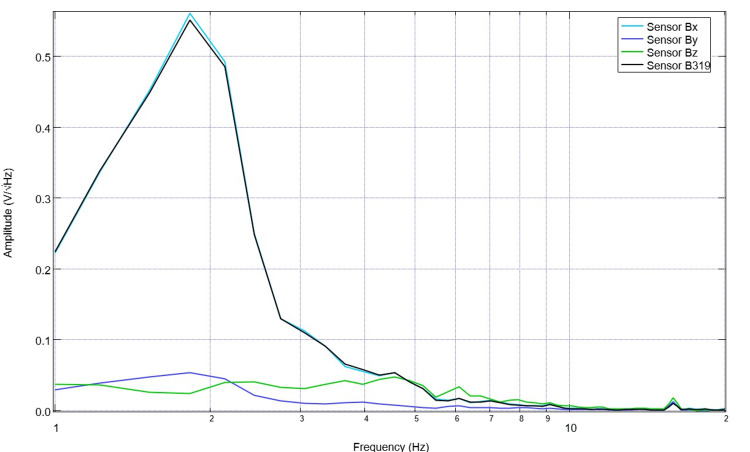
Pre-operative baseline measurements of swine neural circuit-driven electromagnet field while under general anesthesia Cortical electromagnetic fields were measured of the swine subject while under anesthesia prior to controlled cortical impact.

On the day of surgical intervention, baseline noise measurements of the operating suite were obtained (Figure 2). Following this, the pig was anesthetized and then placed in a stereotactic head holder. Once in the head holder, pre-operative EMF measurements were obtained (Figure 3). These pre-operative measurements identified obvious changes from operative room noise and the swine within the head holder. There were noted peaks at 1.8 Hz, 4.5 Hz, 6.2 Hz, 7 Hz, and 9.2 Hz from sensor B319, with valleys at 4.3 Hz, 5.4 Hz, 5.6 Hz, 6.5 Hz, 8.9 Hz, and 10.2 Hz within the swine data.

**Figure 4:**
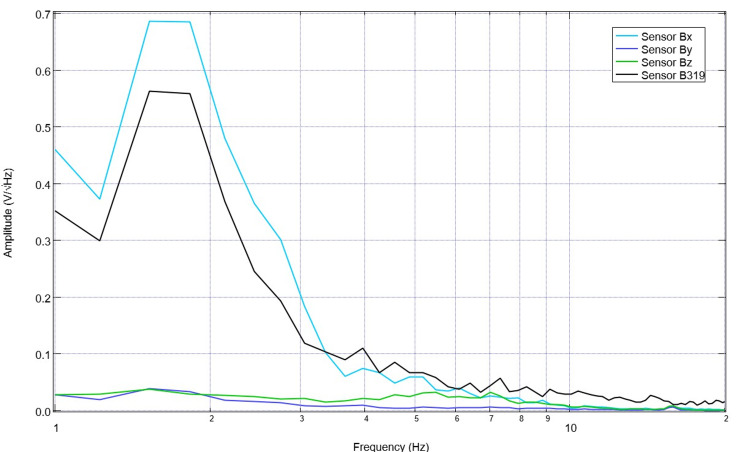
Post-traumatic brain injury electromagnetic field measurements prior to stimulation immediately post-controlled cortical impact while anesthetized Immediate post-controlled cortical impact measurements were obtained of the swine subject while under anesthesia after wound closure. There is a noted plateau beginning at 1.6 Hz and altered morphology compared to pre-operatively.

**Figure 5:**
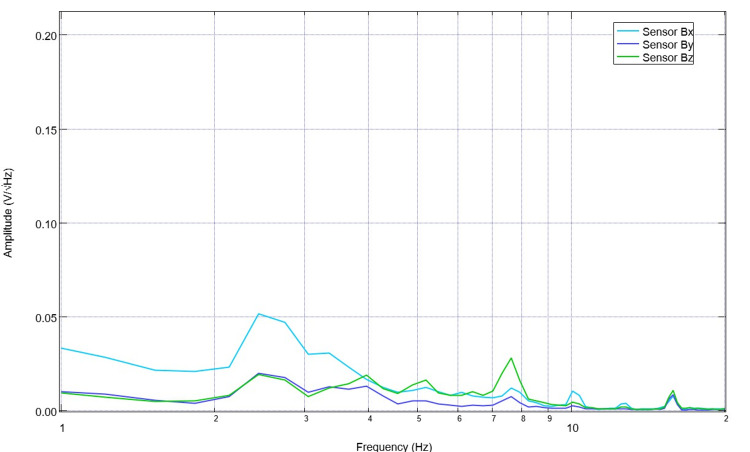
Post-traumatic brain injury electromagnetic field recordings in the subject while anesthetized during stimulation Post-controlled cortical impact electromagnetic field measurements were obtained during stimulation while under anesthesia. Stimulation occurred through a stimulator built into sensor B319 with sensor Bx, By, and Bz recording.

**Figure 6:**
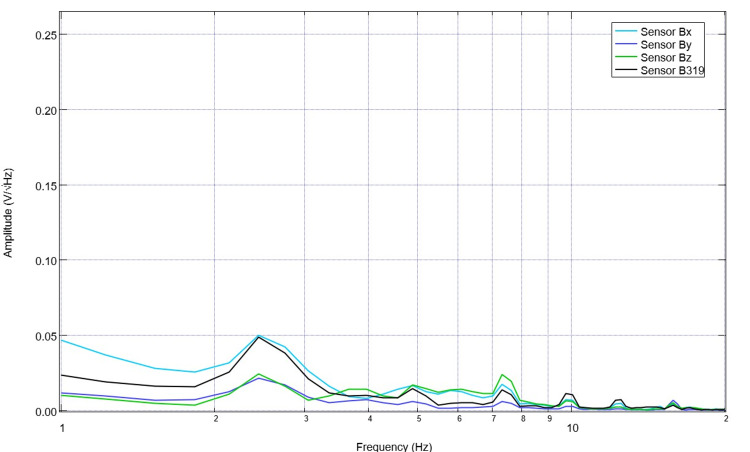
Post-stimulation electromagnetic field recordings of the swine while under general anesthesia Post-stimulation electromagnetic field recordings were obtained of swine cortical circuits while under anesthesia. It is noted that morphologic characteristics were similar to those obtained during stimulation.

After surgical intervention to introduce the lesion through CCI surgery while still under anesthesia, the swine had postoperative EMF measurements obtained (Figure 4). This post-surgical recording exemplifies a plateau that begins at 1.6 Hz and altered peaks and valleys compared to the pre-operative measurements. The peaks immediately post-CCI were identified to be at 1.6 Hz, 1.8 Hz, 3.9 Hz, 4.5 Hz, 6.4 Hz, 7.2 Hz, 8.2 Hz, 9.1 Hz, and 10.5 Hz. Valleys measured by sensor B139 were at 1.2 Hz, 3.6 Hz, 4.2 Hz, 4.8 Hz, 6.1 Hz, 6.7 Hz, 7.6 Hz, 8.9 Hz, and 10 Hz. Following this, the subject was stimulated at 2.5 Hz with a 500mV positive offset set at 1V for two three-minute stimulation trials through sensor B319. During this trial, the sensors Bx, By, and Bz were all simultaneously recorded. Electromagnetic field measurements during this second stimulation trial are noted below in Figure 5. These recordings had an alternative morphology compared to the pre-stimulation measurements. Of note, there was no significant peak identified prior to 2.5 Hz and no well-defined valley at 1.2 Hz. Similarly, a five-minute post-stimulation recording was obtained with similar patterns as those seen during stimulation (Figure 6).

**Figure 7:**
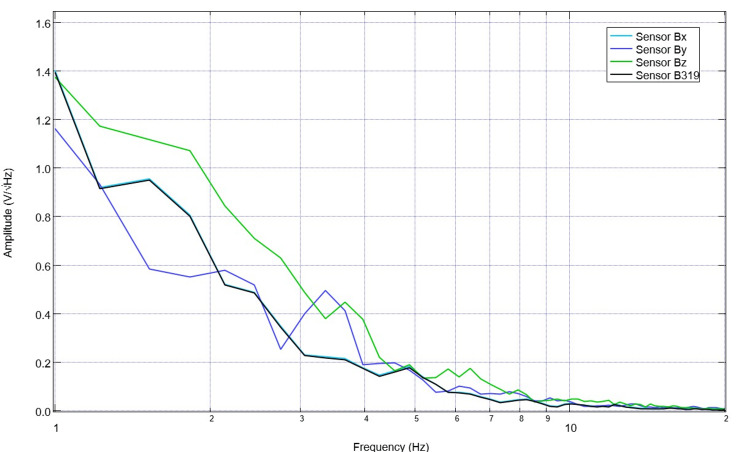
Electromagnetic field measurements after recovery from anesthesia on the day of surgery Electromagnetic field recordings of swine cortical circuits were evaluated post-stimulation upon recovery in the vivarium. The swine had previously undergone two stimulation trials immediately postoperatively.

**Figure 8:**
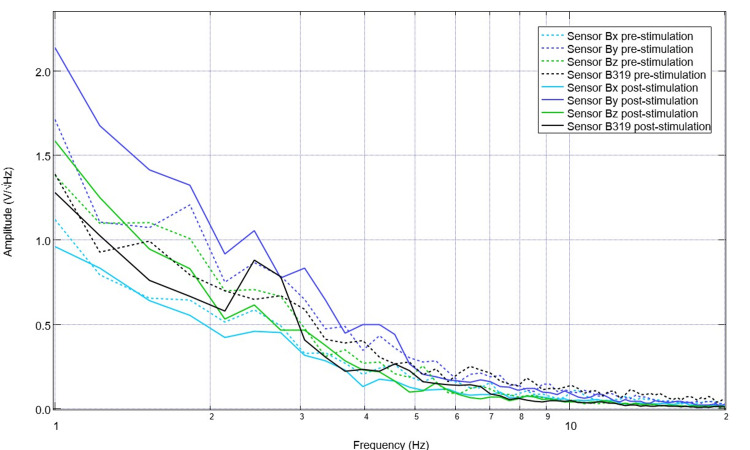
Pre-stimulation and post-stimulation electromagnetic field recordings at 2.5 Hz with 500mV offset at 1V on postoperative day four (stimulation day five) Pre-stimulation and post-stimulation electromagnetic field recordings at 2.5 Hz with 500mV offset at 1V on postoperative day four (stimulation day five) were graphed. The morphology of the waves is noted to be different, especially in wave B319 located over the lesion from controlled cortical impact.

Upon recovery from anesthesia on the day of surgery postoperatively, the swine was evaluated, and post-stimulation and post-surgical EMF recordings were obtained (Figure 7). It is noted that in post-stimulation assessment, there were more defined peaks and valleys compared to those identified while under general anesthesia. Peaks within this immediate post-surgical post-stimulation data were identified at 1.7, 3.6, 4.9, 8.2, and 10Hz, with valleys at 1.4, 2.2, 3.1, 4.3, 5.8, 7.2, and 9.5 Hz. Stimulation occurred once daily for 10 total sessions with settings of 2.5 Hz, 500mV offset at 1 V. Pre-stimulation and post-stimulation neural circuit-generated EMF measurements were recorded each day and compared. A representative pre-and post-stimulation assessment from postoperative day four is noted in Figure 8. It is noted on postoperative day four that when comparing sensor B319 pre-stimulation and post-stimulation, there are morphologic differences between the waveforms. As an example, there is a peak at 2.5 Hz post-stimulation where there was a valley identified at 2.5 Hz pre-stimulation, there is a new valley at 2.2 Hz post-stimulation, and the peak at 1.6 Hz pre-operatively becomes a valley postoperatively. Similarly, it is noted that pre-stimulation, there is a valley at 5.5 Hz.

**Figure 9:**
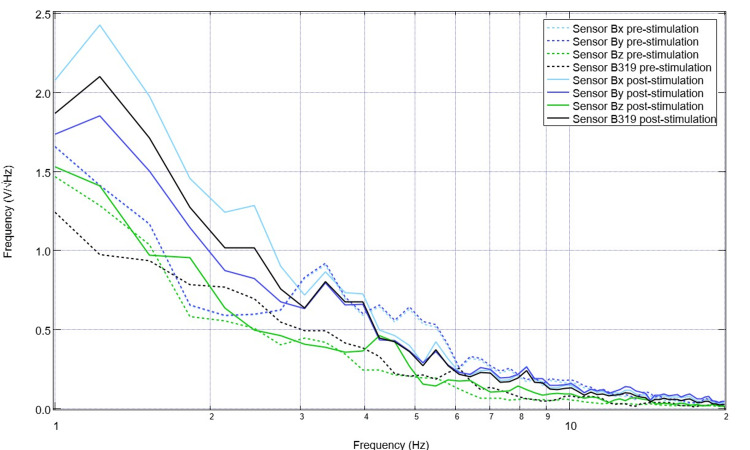
Electromagnetic field measurements prior to and after stimulation with settings of 5.5 Hz, 500mV offset at 1 V on postoperative day 14 (stimulation day 11) Electromagnetic field measurements of the swine subject prior to and after stimulation with settings of 5.5 Hz, 500mV offset at 1 V on postoperative day 14 (stimulation day 11) were recorded. Prior to stimulation on postoperative day 14, 10 days of stimulation were completed at 2.5 Hz with 500mV offset and 1V. Post-stimulation values reflected in the above graph are noted to be after the stimulation settings changed on postoperative day 14 where the frequency was changed to 5.5 Hz without altering the other settings.

After 10 days of stimulation, settings were changed to 5.5 Hz, 500mV offset at 1V. Correspondingly, pre-stimulation and post-stimulation EMF measurements were obtained from the swine using the helmet and sensors within EMF channels (Figure 9). Recordings identified more obvious peaks and valleys after stimulation. Similarly, despite the alteration of the frequency to 5.5 Hz, there is a persistent peak at 2.5 Hz and a new peak at 5.5 Hz post-stimulation as compared to a valley at 5.5 pre-stimulation.

**Figure 10:**
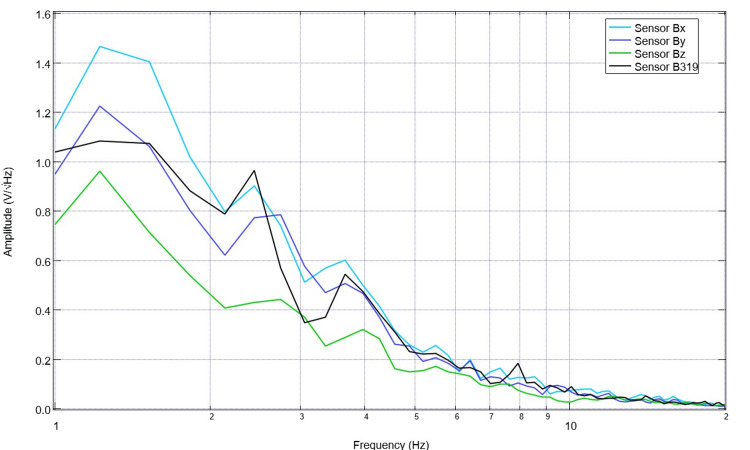
Baseline electromagnetic field recordings from the swine subject on the sixth session of stimulation at 5.5 Hz with a 500mV positive offset at 1 V prior to stimulation Electromagnetic field recordings were obtained for six sessions. The final baseline electromagnetic field recording is identified. It is noted that there were durable changes in the morphology of the waves from prior stimulation trials.

Stimulation occurred at the 5.5 Hz, 500mV offset, and 1 V for six daily sessions prior to sacrifice. The final baseline measurement on this sixth session demonstrates durable change within the morphologic changes from stimulation with a persistent peak at 2.5 Hz; similarly, at 5.5 Hz, there is a persistent peak (Figure 10). This is consistent with the targeted values of stimulation. Baseline daily EMF recordings were then subsequently evaluated using an AI LLM program.

Artificial intelligence software using an LLM model was used to evaluate the baseline EMF measurements of peaks and valleys aggregated from investigators. Data were binned into pre-operative baseline peaks, pre-operative baseline valleys, post-stimulation peaks, and post-stimulation valleys. There were several noted trends identified by the AI LLM model.

In the investigation of the peaks (defined as the positive inflection points), the AI model noted both similarities and differences between the daily data sets. The model identified that there were peaks frequently encountered at 1.8 Hz, 3.9 Hz, 4.5 Hz, 6.4 Hz, 7.2 Hz, and 10.5 Hz. The most frequently identified of these frequencies were 10.5 Hz, seen in 13 measurements, 3.9 Hz in nine measurements, and 6.4 Hz in eight measurements. In evaluating the pre-operative data, there was a unique peak commonly seen at 1.2 Hz compared to the post-stimulation data, where this was only present in the last measurement. The AI model noted that there is variability in the distribution of peaks between frequencies throughout the pre-operative set, with some days having peaks clustered closer together while measurements during other days resulted in more spaced-out peaks. Within the post-stimulation data, 1.6 Hz was a frequently identified peak which was not seen as frequently in pre-stimulation measurements. Like previous studies, there were changes in the distribution of peaks post-CCI [3, 4]. It was found that there were plateaus in peaks post-CCI where-in peaks were divided more among a wider distribution of frequencies along the x-axis and were not as narrowly clustered together after the injury compared to pre-injury evaluation [3,4]. This creates alterations in slope with less variability in change in slope. Importantly, it was noted that on postoperative day 10 (after stimulation treatment 11) that the patterns identified in the B319 sensor measurements, as identified in peaks and valleys, began to resemble pre-CCI patterns. This was defined through the identification of similar patterns of repeated values, intervals between peaks, and similar sequences. This is earlier than in previously published data sets which identified recovery on postoperative day 17 [4]. This recovery was seven days earlier than previously recorded [4]. It was also identified that the 2.5 Hz peak was still present during the final EMF measurement obtained in the trial occurring on postoperative day 21. This identifies durability in the treatment with persistent EMF recovery from stimulation at this threshold.

Within the valleys, there were also observed patterns by the AI model. It identified that within the post-stimulation data set, the distribution of valleys expanded with valleys seen in frequencies of 11 and 11.5Hz, which were not identified pre-operatively. This was suggested by the AI to be changed potentially due to intervention. Within the pre-operative and post-stimulation data sets, valleys located at 6.1 Hz, 4.6 Hz, and 9.2 Hz were noted. The AI model noted that the patterns overall between the data sets were not similar; however, there was an important caveat. Toward the end of the data set, the AI model was queried and noted recovery in patterns of valleys within the swine model. The AI noted that the peaks began to and continued to represent patterns seen in pre-operative data beginning on postoperative day 17 (stimulation day 14). This differs from previously published data where AI convergence in patterns within the valleys was not reported [4].

Behavioral evaluation

The swine was monitored pre-operatively and postoperatively for changes in behavior and activity. Careful attention was taken towards ambulatory capacity, prandial activity, and for signs of post-TBI depression. It was identified that in the post-procedure period, the swine had evidence of ataxia and difficulties with ambulation when recovering within the pen on postoperative day 0. Post-anesthesia, the swine did have evidence of nausea with an episode of vomiting. On postoperative days one and two, the swine no longer had any additional episodes of vomiting but had some difficulty in gait with its right hind leg. Beginning on postoperative day three, the swine no longer exhibited difficulties with ambulation. When considering prandial behaviors, the swine demonstrated a normal appetite every day beginning on postoperative day one. It was observed that the swine appeared more interested in treats used to obtain measurements post-CCI than prior to the intervention. In fact, prior to the intervention, this swine model was not very interested in treats, and it would take several attempts with treats to get the swine's attention. This was changed immediately postoperatively on postoperative day one once nausea resolved when the subject became extremely food motivated and compliant with obtaining measurements. It was also more compliant post-intervention compared to prior to intervention. It was also noted that the swine appeared to exhibit higher levels of activity and impulsivity post-surgery. This was demonstrated through increased activity within the pen when observers were in the room with increased running and climbing on the pen itself. Physiologically, during the surgical intervention, blood pressure was monitored to evaluate whether pre-impact and post-impact blood pressure had an expected increase. Within this subject, it was noted that blood pressure did not change significantly with blood pressure measurements of 128/32 mmHg pre-impact to 124/30 mmHg post-impact. Of note, in this subject, there was hypotension pre-operatively with blood pressure 71/39 mm/Hg and with low diastolic blood pressures during the entirety of the surgical intervention, which may have affected the physiologic response due to disrupted autoregulation due to the diminished mean arterial pressure.

Histology and biomarkers

**Figure 11:**
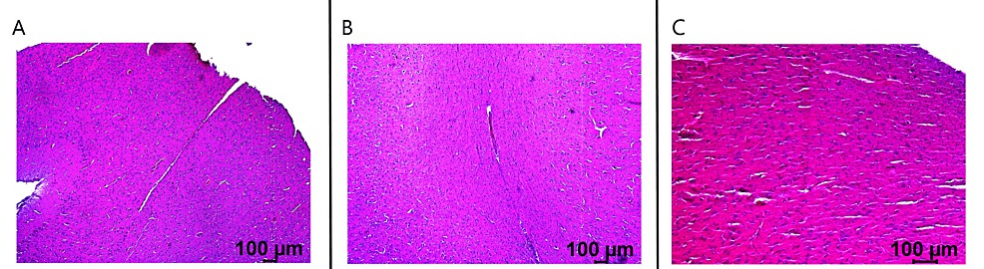
Hematoxylin and eosin staining of cortex in the swine subjects The control swine is seen in panel A, the delayed stimulation swine in panel B, and the immediate stimulation swine in panel C. Samples evaluated are taken directly at the site of controlled cortical impact. It is noted that there is more apoptosis within the control swine compared to the delayed stimulation and immediate EMF stimulation swine. More so, there is more apoptosis in the delayed stimulation swine than in the immediate stimulation swine.

**Figure 12:**
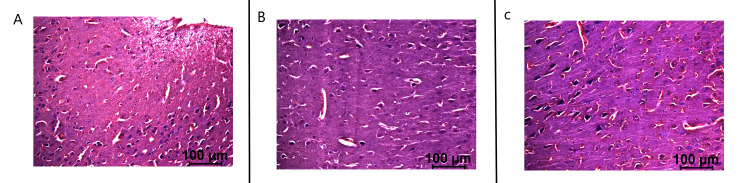
Pentachrome staining of cortex in swine subjects at the site of injury Noted injuries are seen at the site of impact, especially in the control pig. The control swine is seen in panel A, the delayed stimulation swine in panel B, and the immediate stimulation swine in panel C.

Histological specimens were obtained post-euthanasia in the swine specimen. This was compared to specimens obtained from two additional swine that had undergone CCI using the same model [3,4]. The control swine underwent CCI with measurement of cortical EMF without treatment with stimulation. The second swine underwent CCI with stimulation beginning on postoperative day two. The final swine was the swine evaluated in this study which underwent stimulation beginning 20 minutes post-injury while in the operative suite. All swine were sacrificed on postoperative day 21. Histology, as noted through H&E and pentachrome stains, were identified (Figures 11, 12). It is identified that the control swine had more evidence of neural injury than the two EMF-stimulated pigs. This is noted through increased inflammatory infiltrate, apoptotic cells, pyknotic nuclei, and vacuolation. When comparing the delayed stimulation subject to the immediate EMF stimulation subject, it was noted that there was evidence of more viable glial and neural cells and fewer apoptotic cells within the immediate stimulation subject compared to the delayed stimulation subject. Overall, the non-stimulated swine had more apoptotic cells and higher degrees of cellular injury than the stimulated swine.

Neuron-specific enolase measurements were obtained in serum and at the site of injury. Concentrations were assessed. These concentrations are noted below in Tables 1, 2. There are noted differences in NSE concentrations compared to the injured brain and non-injured brain, especially within the stimulation cohort. Additionally, serum NSE values were assessed. Within the delayed stimulation swine, there was a noted increase in NSE concentration immediately post-injury and at sacrifice, with a decrease in NSE concentration on postoperative day 10. The control swine did not demonstrate significant increases in NSE in serum throughout the study. The early stimulation swine demonstrated similar pre-and post-injury NSE values on the day of surgery but had a decrease in NSE on postoperative day two. On postoperative day seven, NSE was noted to increase and slowly decrease until the day of sacrifice.

**Table 1:** Serum neuron-specific enolase levels by swine and date

Swine	Pre-controlled cortical impact neuron-specific enolase concentration (ng/mL)	Post-controlled cortical impact neuron-specific enolase concentration (ng/mL)	Postoperative day 2 neuron-specific enolase concentration (ng/mL)	Postoperative day 7-10 neuron-specific enolase concentration (ng/mL)	Day of sacrifice (postoperative day 21) neuron-specific enolase concentration (ng/mL)
Control		-0.171	-0.105	-0.127	-0.319
Late stimulation (stimulated on postoperative day 2)	0.170	0.307		0.054	0.813
Early stimulation (stimulated 20 minutes after injury)	0.148	0.120	0.016	0.379	0.203

**Table 2:** Tissue neuron-specific enolase levels by swine and location

Swine	Neuron-specific enolase concentration (ng/mL)
Control swine cortex site of controlled cortical impact	0.715
Control swine cortex normal cortex	0.772
Delayed stimulation swine cortex site of controlled cortical impact	1.166
Delayed stimulation swine cortex normal cortex	0.733
Immediate stimulation swine cortex site of controlled cortical impact	1.193
Immediate stimulation swine cortex normal cortex	0.764

## Discussion

Neurological injuries are complex pathologies with limited subcortical assessment of neurologic function, especially in real-time at the circuit level. Real-time, continuous non-invasive evaluation of cortical and subcortical neural circuits could prove invaluable clinically to assess the functioning of critical structures that are responsible for wakefulness, motor function, speech, and other physiologically relevant functions of life. Traumatic brain injury is a well-documented neurological injury known to cause disruption in these circuits and provides a beneficial translational model [3,4]. Furthermore, transcranial magnetic stimulation has undergone investigations to evaluate its effects on neurologic disease, especially in TBI [12,13]. Studies evaluating stimulation have had some encouraging findings for the potential for treatment and even reversal of injury by promoting a healthy EMF environment for the injured neurons to recover and to promote neuroplasticity [7,12,15]. Even more so, EMF has had some evidence of restoration of histological normality post-injury and has been thought to increase growth factor production [8,15]. Even on the subcellular level, EMF has been implicated in microtubule activity and may be associated with appropriate signaling pathways for the transport of critical cellular components [17,18]. Similarly, studies have identified neuroprotective and anti-inflammatory effects of EMF modulation in some models of CNS pathology [19]. Therefore, stimulating and modulating EMF may have profound effects on the cellular and subcellular physiology of neurons and may be a beneficial treatment target for the optimization of abnormal or aberrant neural circuits due to injury, stroke, congenital disease, or other disorders in neural function.

Within large animal studies, swine have begun to be proven to be an appropriate analog for the evaluation of TBI and neurophysiology [1,3,4]. There are appropriate analogous structures with genetic homology. Similarly, Yucatan minipigs have been used previously in early studies to assess the effects of TBI and neural stimulation [3,4]. Similar to other studies, these early studies have shown promise in the effects of targeted neural stimulation on promoting recovery post-TBI. Therefore, continued evaluation of a Yucatan minipig as a translational model is warranted for similar studies.

This investigation identified that post-TBI changes were again reliably seen using a shielded helmet, induction sensors, and shielded EMF channels [3,4]. AI modeling identified distinct differences between pre-operative and postoperative data sets. However, in the case of this study, postoperative data sets were associated with stimulation. In a previous pilot study, the researchers had three distinct categories of pre-operative, postoperative, and post-stimulation EMF measurements using these same sensors and set-up. Within this study, we wished to evaluate the early effects of stimulation to attempt to see if early stimulation would promote early recovery towards pre-operative patterns of EMF. This was done as these sensors and helmets with EMF channels are compact and highly portable. Therefore, there is the potential to utilize this technology in point-of-care settings clinically for hyperacute intervention, diagnosis, and treatment of neurologic injury. Hyperacute interventions may be extremely beneficial in neurologic injury to promote healing and recovery prior to the development of worsened cortical injury from the secondary effects of edema and the inflammatory milieu take effect. Furthermore, due to the portability of these devices, there is the potential for them to be used as an early diagnostic tool in a pre-hospital setting to direct care to more appropriate centers for the degree of injury seen.

Evaluation of the Yucatan minipigs' cortically generated electromagnetic field measurements in this investigation identified differences between pre-operative and post-stimulation data sets. The patterns within these differences were new frequencies measured within peaks and valleys post-stimulation. These patterns represent post-injury and post-stimulation changes generated from signal transduction from neural circuits. Unique to this study, there were slight variations in peaks and valleys compared to previously published literature [3,4]. Similarly, the morphologic patterns in the presented graphs in this study appear to have some differences than in previously published data [3,4]. The unique patterns may be due to variations within individual neural circuits. However, there are some considerable overlaps between these studies. There were noted to be conserved sequences between this study and previously published studies, notably a peak at 2.5 Hz [3,4], which was the reason for its use as a stimulation frequency. An additional observation regarding the 2.5Hz peak was that this is a stimulated threshold, and there was the persistence of this peak late into the trials despite cessation of stimulation at this threshold. This identifies durability in treatment response with immediate stimulation. Despite the differences in peaks and valleys, there were some quantitative similarities with similar peaks (such as 10.5 Hz and 3.9 Hz) and valleys in pre-operative and post-stimulation data sets. These similarities may represent important areas of conserved function in this measured swine. Similarly, these conserved peaks may represent regions where stimulation preserved function as they are identified both pre-stimulation and post-stimulation. Within previous studies, this degree of preserved peaks with 10.5 Hz occurring 13 measurements, 3.9 Hz nine times, and 6.4 Hz seen eight times total in both pre-and post-stimulation measurements was not seen. This may identify a treatment effect.

There were qualitative similarities between the graphical patterns presented in this study with previous studies [3,4]. This includes noted plateaus post-injury prior to electrophysiologic recovery, a decrease in the number of peaks postoperatively, and alterations in slope with less abrupt changes between peaks and valleys. These changes in slope may represent normal physiologic activity and should be investigated further. These patterns will need further investigation and modeling with numerous "normal" and "abnormal" controls to develop appropriate models to correlate with physiologic states. Additionally, these similarities identify both feasibility and reproducibility within this translational model. 

Importantly, it was noted that earlier recovery toward postoperative patterns was identified by AI modeling in EMF measurements compared to prior studies. It was noted that the swine model had recovery in EMF patterns on postoperative day 10 with immediate stimulation compared to post-day 17 with two days of delayed stimulation. This earlier recovery allows for several mechanisms to be theorized in regards to recovery. First off, with stimulation, an appropriate EMF milieu may be created in the region of the injured cells. This may promote the dendrites and axons in cells that were not irreversibly injured to continue to participate in appropriate signaling and processes. Due to this appropriate stimulus in the region and continued activities, the appropriate cellular processes are maintained, allowing for cells to avoid apoptosis and degeneration down an abnormal pathway. Additionally, alterations in the inflammatory process may prevent continued injury or secondary injury [5,19]. Similarly, potential effects on microtubules may promote beneficial signaling patterns post-injury and act as a preventative mechanism [18].

From a behavioral perspective, it was identified that swine had altered neurological functioning post-CCI. This is consistent with evidence of traumatic brain injury with increased impulsivity as measured through climbing within the pen and increased activity. Furthermore, the swine was more interested in treats post-stimulation compared to pre-operatively. This swine also had additional signs of TBI with nausea and vomiting post-procedure, which resolved and is frequently encountered post-TBI.

Histologically, it was identified that there were differences in viable cells comparing the control sample to both stimulated swine, notably with the most cellular viability seen within the early stimulation swine. This identifies the preservation of appropriate cellular processes and, in fact, a restorative effect of EMF on neural cells. This histological recovery is encouraging and identifies a potential treatment paradigm in traumatic brain injury that necessitates further study. In the evaluation of the biomarker analysis, notably, there are increases in NSE concentration within stimulated tissues. This effect was not well demonstrated within the non-stimulated sample despite the increased degree of histological injury identified. This may be due to tissue NSE levels being obtained at postoperative day 21 when an irreversible neurologic injury has already occurred. At that point, there may not have been any more injured cells releasing NSE, and therefore, levels had already reached a nidus. In the stimulated cohort, there was evidence of preserved function and increased cellular viability in a histological sense. The EMF stimulus may have preserved more injured cells resulting in an overall higher concentration of NSE. Alternatively, NSE has complex biological functions and, although it can be frequently implicated in neuronal disease, has also been seen to be involved in neurite regeneration through PI3K/Akt and MAPK/ERK pathways [20]. The overall cellular mechanisms of these noted protective effects of EMF will require additional studies. Despite this, these cellular and EMF findings remain significant and represent critical areas for further investigation. A table correlating the EMF findings with cellular findings and proposed pathophysiological explanations is seen below (Table 3).

**Table 3:** An overall summary of large-scale artificial intelligence-identified patterns with proposed physiologic explanation

Category	Finding	Explanation
Peaks	More spaced-out peaks initially in the postoperative post-stimulation data	Stunned cellular circuits from neural injury
Peaks	Less variation in changes in slope initially in the postoperative post-stimulation data	Stunned cellular circuits from initial neural injury
Peaks	Normalization of electromagnetic field patterns to those seen pre-operatively beginning on postoperative day 10	Preservation of neural connections and early healing through glial involvement and reduction of apoptosis
Peaks	Durability of electromagnetic field changes from stimulation	Preservation of neural connections and development of targeted neural plasticity based on stimulation threshold
Valleys	More spaced-out peaks postoperatively	Stunned cellular circuits from neural injury
Valleys	Normalization of electromagnetic field patterns to those seen pre-operatively beginning on postoperative day 17	Preservation of neural connections and early healing through glial involvement and reduction of apoptosis

From a technical perspective, binned 20-second data sets generate 100,000 data points/Hz. During trials, a 20-second bin was selected; however, trials consisted of several hundred seconds of measurements to obtain appropriate data where the swine was not moving. In a summative fashion, there is now rapid development with exponential amounts of neurophysiologic data gathered from each sensor in the swine model if each 20-second bin or alternative bins are investigated. This physiologic data may be applied in the future to develop appropriate models for physiologic states and for the optimization of treatment. Additionally, from a technical perspective, this trial consisted of an evaluation using four sensors. Increasing the number of sensors and altering angles may allow for subcortical triangulation and evaluation of hyper-specific portions of cellular circuits by investigation of differential portions of EMF measurements and circuitry.

It is noted that stimulation in this trial occurred using previously published settings. Further studies may wish to take alternative approaches. As it was identified that there are some unique patterns to the individual subject that are modified postoperatively from CCI, rapid evaluation of post-injury changes may be needed to attempt to better target stimulation thresholds and settings. However, in order to do so, a model of "normal" EMF patterns must be constructed from modeling using a large sample of healthy subjects. From this, models of TBI can be created using post-impact injury when using this translational model. Further studies with large samples may utilize advanced AI learning algorithms to better construct these models to understand these post-injury findings identified in this study and previous studies [3,4]. Furthermore, within the EMF signal generator, there are numerous settings to evaluate, and with larger sample sizes, alternative settings can be investigated to ensure appropriate targeting and treatment of these lesions to attempt to identify optimum strategies for reversing electromagnetically demonstrated injury. Similarly, histological and serological markers may be investigated to evaluate the effects of targeted EMF in treatment and to evaluate if stimulatory treatment causes histologic recovery as was previously demonstrated in rat studies using analogous treatment with TMS [8]. 

Limitations

This study was designed as a pilot study with a single individual as a sample size to evaluate the feasibility of early stimulation of cortical electromagnetic fields. Therefore, larger studies are needed to confirm the reproducibility of these findings. Similarly, LLM AI modules are prone to hallucinations, although these were evaluated prior to reporting. Further studies may require more complex AI algorithms utilizing unsupervised learning techniques and alternative models. Stimulation occurred at previously published thresholds. These thresholds may not have been ideal targets for the individual TBI in this swine. As each TBI has unique characteristics with subjects containing unique degrees of physiology and variable anatomy, using rapid interpretation of postoperative EMF changes may be necessary to optimize treatment. Furthermore, a bank of normal values similarly may be required in the future to create better models of normal to assess for post-TBI related changes to better target signaling to promote recovery. Nevertheless, there were changes post-stimulation that appeared to optimize the EMF environment with some normality returning to EMF signal patterns. Additional limitations include the usage of only four sensors. With increased sensor utilization, there is the potential for increased spatial and temporal evaluation for more targeted evaluation of subcortical neural circuitry. This identifies the potential for efficacy as a treatment in TBI and neurologic disease and identifies the feasibility of this large animal translational approach for further investigations of identifying normality, abnormalities, pathology, and potentially treatment for neural circuits non-invasively in real-time.

## Conclusions

Neural circuits have been measured in the swine cortex using non-invasive, continuous real-time methods of electromagnetic field measurement with induction sensors isolating shielding to a helmet. Similarly, electromagnetic field stimulation has been identified to be a potential treatment modality for induced traumatic brain injury through a controlled cortical impact model. Hyperacute stimulation, 20 minutes after injury, was identified to have earlier and durable electrophysiologic recovery to baseline pre-injury patterns of neuronal EMF in this trial compared to a prior trial of targeted EMF. Similarly, there is evidence of the neuroprotective and regenerative effects of EMF through histological recovery and decreased level of neural apoptosis and injury in EMF-stimulated swine compared to non-stimulated swine with evidence of a time-dependent effect on cellular viability. The effect of early EMF recovery identifies healing at the cellular, circuit, and subcellular levels, with the introduction of possibilities of additional investigations on modulation and stimulation of EMF for treatment in neurological disease. This identifies an area of promise for further study for new avenues of treatment in additional populations, including hyperacute treatment of traumatic brain injury patients, and for evaluation of the application of this modality in neurologic diseases, including stroke, as well as neural degenerative disorders such as Alzheimer's disease. 
